# Do Copy Number Changes in *CACNA2D2*, *CACNA2D3*, and *CACNA1D* Constitute a Predisposing Risk Factor for Alzheimer’s Disease?

**DOI:** 10.3389/fgene.2016.00107

**Published:** 2016-06-14

**Authors:** Darine Villela, Claudia K. Suemoto, Carlos A. Pasqualucci, Lea T. Grinberg, Carla Rosenberg

**Affiliations:** ^1^Human Genome and Stem-Cell Research Center, Department of Genetics and Evolutionary Biology, Institute of Biosciences, University of São Paulo, São PauloBrazil; ^2^Discipline of Geriatrics, Department of Clinical Medicine, School of Medicine, University of São Paulo, São PauloBrazil; ^3^Brazilian Aging Brain Study Group – LIM22, Department of Pathology, School of Medicine, University of São Paulo, São PauloBrazil; ^4^Department of Pathology, School of Medicine, University of São Paulo, São PauloBrazil; ^5^Memory and Aging Center, Department of Neurology, University of California, San Francisco, San Francisco, CAUSA

**Keywords:** Alzheimer’s disease, copy number variations, CNVs, array-CGH, Ca^2+^

## Abstract

Dysregulation of calcium (Ca^2+^) homeostasis is now being recognized to be a key step in the pathogenesis of Alzheimer’s disease (AD). Data from the literature, in particular the association between AD and polymorphism that interfere with Ca^2+^ homeostasis indicates the presence of genetic factors in this process; further, presenilins mutations, which are known to cause the familial form of AD, are involved in the regulation of intracellular Ca^2+^ stores. Here, we wish to draw attention to rare DNA copy number variations identified in two subjects with late-onset AD that led to partial or full duplication of genes that encode different subunits of the same type of voltage-gated Ca^2+^ channels; these duplications of voltage-gated Ca^2+^ channel genes is consistent with the critical role of calcium signaling in molecular processes underlying memory as has been demonstrated by several studies.

It’s widely known that Ca^2+^ represents one of the most important second messengers in the brain and plays an essential role in neuronal development, synaptic transmission and plasticity, besides regulating various metabolic pathways (Striessnig et al., 2006). The regulation of Ca^2+^ homeostasis in the central nervous system is a very complex process involving proteins localized in the plasma membrane, endoplasmic reticulum (ER), mitochondria, and cytoplasm that together are responsible for maintaining a higher concentration of Ca^2+^ in the extracellular space (Berridge et al., 2003). Notably, as demonstrated by several studies, a dysregulation in Ca^2+^ homeostasis is associated with many pathological mechanisms, especially those related with neurodegenerative disorders (Berridge, 2013; Sulzer and Surmeier, 2013).

Based on previous observations that intracellular Ca^2+^ levels are increased in aging neurons, it has been suggested that a dysregulation of Ca^2+^ homeostasis could be a key step in the pathogenesis of Alzheimer’s disease (AD; Small, 2009). In fact, this is the basis of the calcium hypothesis of AD, which offers a potential correlation between β-amyloid (Aβ) toxicity and a progressive decline in memory and neuronal cell death (Fedrizzi and Carafoli, 2011). The central idea is that activation of the amyloidogenic pathway leads to a remodeling of the neuronal Ca^2+^ signaling, and this remodeling usually appears as upregulated in AD, triggered by either an enhance in the entry of external Ca^2+^ or a release from internal stores (Berridge, 2010).

The Aβ peptide, present in the extracellular senile plaques, is one of the hallmarks of the disease and its aggregation in oligomers has been found to induce Ca^2+^ influx into neurons functioning as channels and/or by activating channels in the plasma membrane (Demuro et al., 2010). The Aβ oligomers could form non-selective pores with high conductance for Ca^2+^ but they could also alter the fluidity of the membrane affecting the activity of several Ca^2+^ permeable channels, including acetylcholine receptors, glutamate receptors, dopamine receptors, and serotonin receptors (Eckert et al., 2005). On the other hand, within the neurons, Aβ interacts with two types of Ca^2+^ channels in the ER membrane that regulate calcium release from internal stores: (i) ryanodine receptors (RyRs) and (ii) inositol trisphosphate receptors (IP3Rs); this leads to an increase of their opening and the amount of Ca^2+^ being released by the ER (Chakroborty et al., 2009). It is relevant to note, though, that data from literature demonstrate that genetic factors may be crucial in the dysregulation of Ca^2+^ homeostasis in AD. Presenilins mutations, which are known to cause the familial form of AD, have been found to increase the release of intracellular Ca^2+^ from internal stores (Honarnejad and Herms, 2012; Bezprozvanny, 2013). The activity of the SERCA pump, which is responsible for maintain the Ca^2+^ concentration gradient across the ER membrane (higher inside) is enhanced by the presenilins (Green et al., 2008). Additionally, there is increasing evidence that presenilins may be leak channel. Tu et al. (2006) reported that the mutated form of presenilin 1 (*PS1*) reduces the passive leak in neurons resulting in an increase of cytoplasmatic Ca^2+^.

Of particular interest, it was demonstrated that a polymorphism in *CALHM1* (Ca^2+^ homeostasis modulator-1) was significantly associated with the sporadic form of the disease in a case-control study (Dreses-Werringloer et al., 2008). Based on its sequence similarity to the ionotropic glutamate receptor NMDA, it has been proposed that this gene encodes a glycoprotein that is a cerebral Ca^2+^ channel component, which is localized both in the plasma membrane and ER, and may also function as a leak channel (Dreses-Werringloer et al., 2008). The expression of *CALHM1* polymorphic variant reduces Ca^2+^ entry and this reduced Ca^2+^ permeability would increase the level of stored Ca^2+^ by reducing this putative leak pathyway; consequently, a decreased intracelullar Ca^2+^ levels is observed (Dreses-Werringloer et al., 2008). The authors also suggest a direct impact of *CALHM1* on Aβ production. Although some studies argue against the latter observation, it seems that the problem is the different methods used for analysis of the data (Bertram et al., 2008; Rubio-Moscardo et al., 2013). Besides, even though it seems controversial that in the familial form of AD an increase of intracellular Ca^2+^ is observed while in the sporadic form studies report a reduce in intracellular Ca^2+^ levels, dysregulation of Ca^2+^ homeostasis in the two forms of AD, as described above, arise by different mechanisms, but what is pretty clear in the literature is that dysregulation of Ca^2+^ homeostasis is a major feature in AD.

More recently, a multicenter study reported a robust and consistently significant enrichment for genes constituting the calcium signaling pathway, especially those related to the elevation of cytosolic calcium, in independent cohorts of young and elderly participants (Heck et al., 2015). The authors also showed that the same gene set identified in those participants was significantly enriched in a very large case-control study of sporadic AD, making evident that calcium signaling is crucial in human memory processes, both in cognitive health and disease (Heck et al., 2015).

In an investigation about the frequency of DNA copy number variations (CNVs) in a cohort of 46 individual’s diagnosed post-mortem with late-onset AD provided by the Brain Bank of the Brazilian Aging Brain Study Group (BBBABSG; Grinberg et al., 2007). We found two cases with rare microduplications partially or fully affecting genes that encode different subunits of the same type of voltage-gated Ca^2+^ channels, the L-type (L-VGCC). One individual presented a genomic imbalance of 665 kb at 3p21.1 that incudes, among other genes, *CACNA1D* and *CACNA2D3* (**Figure 1A**). *CACNA1D* encodes the subunit α1 (subtype _1D_), which form the ion-conducting pore of the L-VGCC, and *CACNA2D3* is responsible for encoding the subunit α-2δ, that influences the channels’ biophysical properties by increasing the current amplitude and regulating the activation and inactivation kinetics of the L-VGCC. On the other hand, the other subject showed a genomic imbalance of 300 kb at 3p21.31 that encompass the *CACNA2D2* gene, which acts similarly to *CACNA2D3*, i.e., also encodes the subunit α-2δ of the L-VGCC (**Figure 1B**). Both duplications have intragenic breakpoints; depending on the resulting structure of the rearrangement, the partial gene duplications can result in loss of function of the genes involved.

**FIGURE 1:**
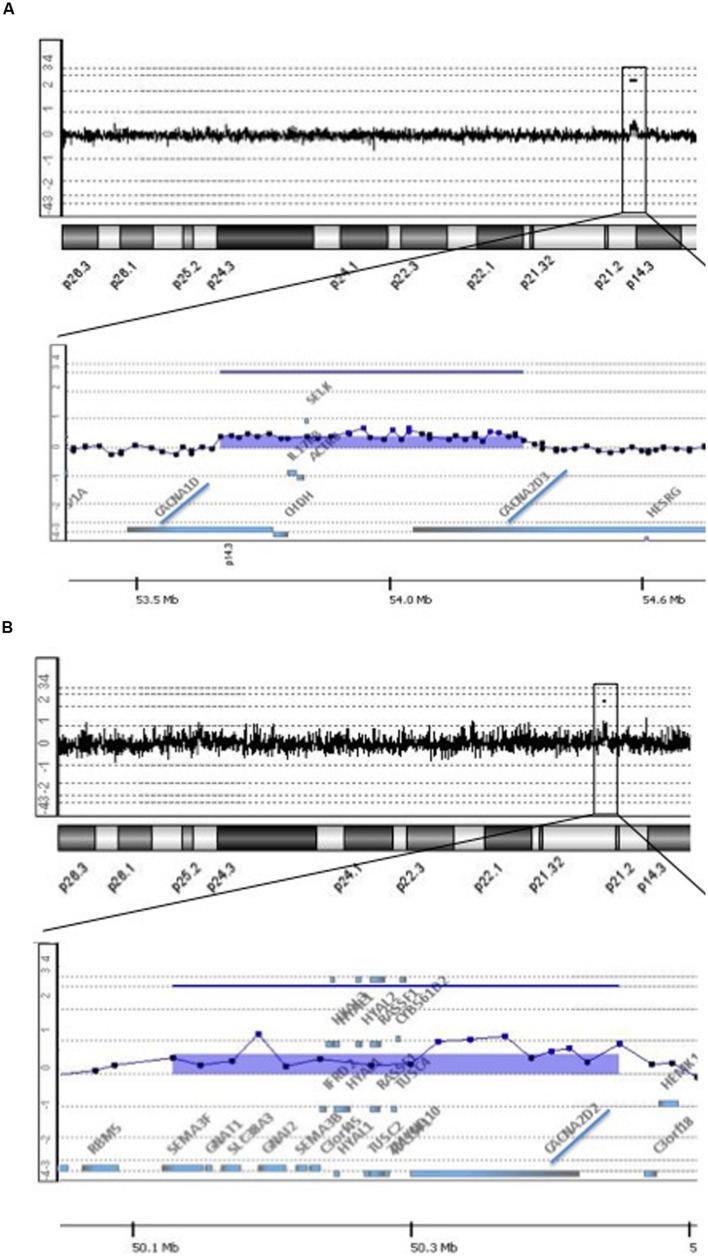
**Rare copy number variations (CNVs) implicated in voltage gate calcium channels detected in two individuals with late-onset Alzheimer’s disease.** The figure shows array-CGH results where each dot represents a probe. A genomic segment was considered duplicated or deleted when the log_2_ ratio of the Test/Reference fluorescent intensities of a given region encompassing at least three probes was above 0.3 or below -0.3, respectively, (Images were extracted from Genomic Workbench software). **(A)** Top shows array-CGH profile of chromosome 3 of Individual 1; the black box indicates the location of the microduplication on the short arm of the chromosome; underneath a detailed view of the area comprised in the black box at 3p21.1; the image shows a 665 kb duplication containing among other genes, *CACNA1D* and *CACNA2D3*, highlighted by a blue trace. **(B)** Top shows array-CGH profile of chromosome 3 of Individual 2; the black box indicates the location of the microduplication on the short arm of the chromosome; underneath a detailed view of the area comprised in the black box at 3p21.31; the image shows a 300 kb duplication containing among other genes, *CACNA2D2*, highlighted by a blue trace.

To exclude that these rare CNVs (rare defined as frequency <0.1% of population, based on the Database of Genomic Variants – DGV) represent common variants in the Brazilian population, we compile CNV data from more than 1,300 individuals studied by array-CGH in our laboratory for reasons other than dementia, such as cancer predisposition, deafness, congenital abnormalities, and intellectual disability. The rare CNVs documented in this study were never detected in any of these subjects. Additionally, in contrary to the control sample in this study, which consisted in individuals with no anatomophatological signs of AD, this set of more than 1,300 individuals is supposedly similar to the population regarding AD predisposition factors. In particular, despite some of the other individuals investigated in our casuistic also presented rare CNVs, these two cases are the only ones who showed copy number changes in genes that constitute the calcium signaling pathway. However, we cannot exclude that the remaining 44 individuals may have point mutations in relevant genes of calcium signaling pathway that could not be observed due to a limited resolution of array-CGH.

CNVs are recognized to be a prevalent form of common genetic variation and represent a substantial proportion of total genetic variability in human population. The functional impact of CNVs has been demonstrated at all biological levels, from cellular effects on gene expression to their association with several types of complex traits and genetic diseases (Stankiewicz and Lupski, 2010). To date, copy number changes in genes encoding calcium-signaling molecules have been associated especially with neuronal development disorders, such as autism (Rosenfeld et al., 2010), psychiatric diseases (Lee et al., 2012), and amyotrophic lateral sclerosis.

The present evidence is the first to highlight genomic imbalances in genes that are known to encode important calcium-signaling molecules, and link those CNVs to late-onset AD. Of note, in the manuscript from Heck et al. (2015) it was demonstrated that the same gene family of *CACNA1D*, *CACNA2D2*, and *CACNA2D3* was found to be significantly enriched in patients with AD, which reinforces the significance of our findings. The present data also contribute adding new mutations in genes of calcium signaling pathway and associate these mutations with AD since *CACNA1D*, *CACNA1D*, *CACNA2D2*, and *CACNA2D3* are not in the list of genes studied by Heck et al. (2015). Thus, considering the importance of L-VGCC in the central nervous system and Ca^2+^ being such an important regulator of synaptic plasticity, we could suppose that a disruption in *CACNA1D*, *CACNA2D3*, and *CACNA2D3* caused by CNVs may lead to a dysregulation in Ca^2+^ homeostasis and contribute to the pathogenesis of AD. Also, using the Variant Effect Predictor (VEP) tool from Ensemble^1^ to predict the effects of these two microduplications on genes, transcripts, and protein sequence, as well as regulatory regions, we found that the most likely consequence (34%) is amplification of the amount of transcript. Therefore, this prediction suggests a possible upregulation of Ca^2+^ signaling, which meets the central idea of the calcium hypothesis of AD (Berridge, 2010). Yet, the crucial question of whether the microduplications showed in this study constitutes a predisposing risk factor for AD can only be answered by similar investigations in large cohorts, which we hope will be stimulated by this report. Also, we believe that the present evidence may stimulate further functional studies and eventually confirm the impact of these copy number changes in the pathogenesis of AD.

In summary, we wish to draw attention to rare DNA CNVs identified in two subjects with late-onset AD that led to partial or full duplication of genes that encode different subunits of the same type of voltage-gated Ca^2+^ channels and point out that the present finding is consistent with the critical role of calcium signaling in molecular processes underlying memory as has been demonstrated by several studies.

## Author Contributions

This study was coordinated by CR and DV, as the principal investigator, provided conceptual and technical guidance for all aspects of this study. CS, LG, and CP coordinate the Brain Bank of the Brazilian Aging Study Group and provided all the samples. This commentary was written by DV.

## Conflict of Interest Statement

The authors declare that the research was conducted in the absence of any commercial or financial relationships that could be construed as a potential conflict of interest.

## Footnotes

1 http://www.ensembl.org/info/docs/tools/vep/index.html

## References

[B1] Berridge M. J. (2010). Calcium hypothesis of Alzheimer’s disease. *Pflugers. Arch.* 459 441–449. 10.1007/s00424-009-0736-1 19795132

[B2] Berridge M. J. (2013). Dysregulation of neural calcium signaling in Alzheimer disease, bipolar disorder and schizophrenia. *Prion* 7 2–13. 10.4161/pri.21767 22895098 PMC3609045

[B3] Berridge M. J. Bootman M. D. Roderick H. L. (2003). Calcium signalling: dynamics, homeostasis and remodelling. *Nat. Rev. Mol. Cell Biol.* 4 517–529. 10.1038/nrm1155 12838335

[B4] Bertram L. Schjeide B. M. Hooli B. Mullin K. Hiltunen M. Soininen H. (2008). No association between CALHM1 and Alzheimer’s disease risk. *Cell* 135 993–994; author reply 994–996. 10.1016/j.cell.2008.11.030 19070563 PMC2841134

[B5] Bezprozvanny I. (2013). Presenilins and calcium signaling-systems biology to the rescue. *Sci. Signal.* 6 e24. 10.1126/scisignal.2004296 PMC5018382 23838181

[B6] Chakroborty S. Goussakov I. Miller M. B. Stutzmann G. E. (2009). Deviant ryanodine receptor-mediated calcium release resets synaptic homeostasis in presymptomatic 3xTg-AD mice. *J. Neurosci.* 29 9458–9470. 10.1523/JNEUROSCI.2047-09.2009 19641109 PMC6666542

[B7] Demuro A. Parker I. Stutzmann G. E. (2010). Calcium signaling and amyloid toxicity in Alzheimer disease. *J. Biol. Chem.* 285 12463–12468. 10.1074/jbc.R109.080895 20212036 PMC2857063

[B8] Dreses-Werringloer U. Lambert J. C. Vingtdeux V. Zhao H. Vais H. Siebert A. (2008). A polymorphism in CALHM1 influences Ca2+ homeostasis, Abeta levels, and Alzheimer’s disease risk. *Cell* 133 1149–1161. 10.1016/j.cell.2008.05.048 18585350 PMC2577842

[B9] Eckert G. P. Wood W. G. Muller W. E. (2005). Membrane disordering effects of beta-amyloid peptides. *Subcell Biochem.* 38 319–337. 10.1007/0-387-23226-5_16 15709486

[B10] Fedrizzi L. Carafoli E. (2011). Ca2+ dysfunction in neurodegenerative disorders: Alzheimer’s disease. *Biofactors.* 37 189–196. 10.1002/biof.157 21698698

[B11] Green K. N. Demuro A. Akbari Y. Hitt B. D. Smith I. F. Parker I. (2008). SERCA pump activity is physiologically regulated by presenilin and regulates amyloid beta production. *J. Gen. Physiol.* 181 1107–1116. 10.1085/JGP1322OIA1 18663130

[B12] Grinberg L. T. Ferretti R. E. Farfel J. M. Leite R. Pasqualucci C. A. Rosemberg S. (2007). Brain bank of the Brazilian aging brain study group - a milestone reached and more than 1,600 collected brains. *Cell Tissue Bank.* 8 151–162. 10.1007/s10561-006-9022-z 17075689

[B13] Heck A. Fastenrath M. Coynel D. Auschra B. Bickel H. Freytag V. (2015). Genetic analysis of association between calcium signaling and hippocampal activation, memory performance in the young and old, and risk for sporadic alzheimer disease. *JAMA Psychiatry* 72 1029–1036. 10.1001/jamapsychiatry.2015.1309 26332608 PMC5291164

[B14] Honarnejad K. Herms J. (2012). Presenilins: role in calcium homeostasis. *Int. J. Biochem. Cell Biol.* 44 1983–1986. 10.1016/j.biocel.2012.07.019 22842534

[B15] Lee K. W. Woon P. S. Teo Y. Y. Sim K. (2012). Genome wide association studies (GWAS) and copy number variation (CNV) studies of the major psychoses: what have we learnt? *Neurosci. Biobehav. Rev.* 36 556–571. 10.1016/j.neubiorev.2011.09.001 21946175

[B16] Rosenfeld J. A. Ballif B. C. Torchia B. S. Sahoo T. Ravnan J. B. Schultz R. (2010). Copy number variations associated with autism spectrum disorders contribute to a spectrum of neurodevelopmental disorders. *Genet. Med.* 12 694–702. 10.1097/GIM.0b013e3181f0c5f3 20808228

[B17] Rubio-Moscardo F. Seto-Salvia N. Pera M. Bosch-Morato M. Plata C. Belbin O. (2013). Rare variants in calcium homeostasis modulator 1 (CALHM1) found in early onset Alzheimer’s disease patients alter calcium homeostasis. *PLoS ONE* 8:e74203 10.1371/journal.pone.0074203 PMC3775809 24069280

[B18] Small D. H. (2009). Dysregulation of calcium homeostasis in Alzheimer’s disease. *Neurochem. Res.* 34 1824–1829. 10.1007/s11064-009-9960-5 19337829

[B19] Stankiewicz P. Lupski J. R. (2010). Structural variation in the human genome and its role in disease. *Annu. Rev. Med.* 61 437–455. 10.1146/annurev-med-100708-204735 20059347

[B20] Striessnig J. Koschak A. Sinnegger-Brauns M. J. Hetzenauer A. Nguyen N. K. Busquet P. (2006). Role of voltage-gated L-type Ca2+ channel isoforms for brain function. *Biochem. Soc. Trans.* 34 903–909. 10.1042/BST0340903 17052224

[B21] Sulzer D. Surmeier D. J. (2013). Neuronal vulnerability, pathogenesis, and Parkinson’s disease. *Mov. Disord.* 28 715–724. 10.1002/mds.25187 23589357

[B22] Tu H. Nelson O. Bezprozvanny A. Wang Z. Lee S. F. Hao Y. H. (2006). Presenilins form ER Ca2+ leak channels, a function disrupted by familial Alzheimer’s disease-linked mutations. *Cell* 126 981–993. 10.1016/j.cell.2006.06.059 16959576 PMC3241869

